# Dysphagia in patients with moderate and severe obstructive sleep apnea

**DOI:** 10.1016/j.bjorl.2019.10.004

**Published:** 2019-11-15

**Authors:** Milena de Almeida Torres Campanholo, Fabio de Azevedo Caparroz, Renato Stefanini, Leonardo Haddad, Lia Rita Azeredo Bittencourt, Sergio Tufik, Fernanda Louise Martinho Haddad

**Affiliations:** a Universidade Federal de São Paulo (UNIFESP), Escola Paulista de Medicina, Departamento de Otorrinolaringologia e Cirurgia de Cabeça e Pescoço, São Paulo, SP, Brazil; b Universidade Federal de São Paulo (UNIFESP), Escola Paulista de Medicina, Departamento de Psicobiologia, São Paulo, SP, Brazil

**Keywords:** Swallowing disorders, Obstructive sleep apnea, Dysphagia

## Abstract

**Introduction:**

There is evidence that trauma caused by snoring in the pharynx could result in dysphagia in patients with obstructive sleep apnea, but the literature is still scarce to define the factors associated with the presence of dysphagia in these patients.

**Objectives:**

To analyze the occurrence of dysphagia and its clinical and polysomnographic features in patients with moderate and severe obstructive sleep apnea, in addition to verifying the impact of dysphagia on the quality of life of these patients.

**Methods:**

Seventy patients with moderate or severe apnea (apnea and hypopnea index – AHI > 15/hour) were selected. The patients underwent a sleep questionnaire, a quality of life in dysphagia questionnaire and a fiberoptic endoscopic evaluation of swallowing.

**Results:**

A total of 70 patients were included in the study, of which 49 were men (70 %), with a mean age of 48.9 years. The fiberoptic endoscopic evaluation of swallowing was altered in 27.3 % and the most frequent alteration was the premature oral leakage with fluid. Comparing the groups with and without dysphagia, the female gender was the only clinical parameter that showed a trend of statistical significance in the group with dysphagia (*p* = 0.069). There was no statistical difference regarding the polysomnographic features and in the global quality of life score in dysphagia in the comparison between the groups.

**Conclusions:**

The presence of dysphagia in patients with moderate to severe apnea is frequent and subclinical, reinforcing the need to investigate this symptom in this group of patients. However, the presence of dysphagia did not result in worsening in patients' quality of life, suggesting that, although frequent, its effect is mild. There was no relevance regarding the association of clinical and polysomnographic parameters with the presence of dysphagia.

## Introduction

The pharynx is a multifunctional organ, common to the airway and gastrointestinal tract, integrating complex activities such as breathing and swallowing. To ensure that breathing and food intake can occur adequately, without adverse effects from one tract to another, effective neural control is required, integrating sensory receptors and effector muscles. During wakefulness, the airway remains patent due to the intense activity of the pharyngeal dilator muscles; however, after sleep onset, the muscle activity is reduced, favoring airway collapse.^1^ In patients with Obstructive Sleep Apnea (OSA), the balance between negative pressure in the pharyngeal lumen and the pressure exerted by the pharyngeal dilator muscles, due to multifactorial causes, is not effectively preserved during sleep, favoring pharyngeal collapse.

Patients with OSA and primary snoring have repetitive pharyngeal collapse events and vigorous vibration caused by snoring. These vibratory factors cause trauma to the upper airway (UA) mucosa, resulting in edema and local neurological damage.^2^, ^3^ Considering the importance of stimulating the pharyngeal sensory receptors in the swallowing process for bolus preparation, adequate positioning of oropharyngeal structures, modulation of muscle contraction strength, velocity and time studies in the literature suggest that the vibratory trauma caused by OSA and snoring could affect the adequate swallowing function,^4^ which could have an additional impact on the worsening of quality of life in these patients.

Considering the importance of pharyngeal sensitivity integrity during the swallowing process, the presence of swallowing abnormalities in OSA patients is expected, as studies have shown. Initially Teramoto et al., in 1999, demonstrated a delay in the swallowing reflex onset.^5^ In another study using videofluoroscopy, in patients without dysphagia complaints, a swallowing alteration was observed with premature escape of the pharyngeal bolus and a delay in the evocation of the swallowing reflex.^6^ In 2011, Valbuza et al. carried out a pilot study, assessing swallowing through videoendoscopy, evaluating 11 patients with moderate and severe OSA without dysphagia and 14 subjects without snoring or apnea in the control group. Premature oral leakage occurred in 64 % of patients with moderate and severe OSA.^7^

Although previous studies indicate that patients with obstructive sleep apnea have dysphagia-related subclinical manifestations, the results are still limited in the literature on which clinical and polysomnographic findings could be associated with the presence of dysphagia in this group of patients, as well as the additional impact of dysphagia on the quality of life of OSA patients.

The aim of this study was to evaluate the clinical and polysomnographic profile of dysphagia in patients with moderate and severe obstructive sleep apnea associated with the assessment of the additional impact of dysphagia on the quality of life of patients with moderate and severe OSA.

## Methods

A prospective study was performed at the Sleep Disorders Outpatient Clinic of the Department of Otorhinolaryngology and Head and Neck Surgery of Universidade Federal de São Paulo-UNIFESP/EPM, from November 2014 to December 2016. The project was approved by the Research Ethics Committee (REC) of this institution - Plataforma Brasil, according to CAAE Opinion n. 44406715.6.0000.5505 and all included patients signed the Free and Informed Consent Form.

During this period, 70 individuals aged 18–65 years, of both genders, who had a polysomnographic assessment compatible with moderate and severe obstructive sleep apnea detected by full-night polysomnography performed at the Sleep Laboratory of the Outpatient Clinic of Sleep Disorders of the Department of Otorhinolaryngology and Head and Neck Surgery of Universidade Federal de São Paulo - UNIFESP/EPM, during a maximum period of 6 months prior to the study (OSAS > 15 events/hour ‒ ICDS 2005) were included in the study.^8^

Patients on gastric medication on the previous 30 days, users of psychoactive substances and sedative medications, alcoholic patients, and those with decompensated clinical or psychiatric diseases were excluded. Patients that had previously used a positive pressure device were also excluded, as well as individuals older than 65 years (to reduce the risk of presbyphagia) and patients with a history of head and neck tumors or neurological disease.

These patients underwent the research protocol with the Epworth Sleepiness Scale (ESS) questionnaires,^9^ SWAL-Qol (quality of life questionnaire in dysphagia)^10^, ^11^ and swallowing videoendoscopy.^12^, ^13^ Epidemiological and anthropometric data were collected from all patients, as follows: age, gender, BMI and neck circumference.

The SWAL-Qol questionnaire consists of 44 questions divided into 11 diet-related quality of life domains (burden, duration, appetite, symptom frequency, food selection, fear, mental health, social life, sleep and fatigue). The possible answers are: “always” (0 points), “often” (25 points), “sometimes” (50 points), “rarely” (75 points) and “never” (100 points).

The scoring for each domain is obtained by adding the scores of the answers to the questions and dividing this by the number of questions in each domain. The score for each domain can range from 0 (worst) to 100 (best). The Portuguese version of SWAL-Qol, translated and validated by Montoni and Alves,^11^ was used in this study.

Regarding the swallowing videoendoscopy, a baseline evaluation was performed, verifying possible anatomical alterations, velopharyngeal closure, presence of salivary stasis, laryngeal sensitivity and vocal fold mobility. In the dynamic evaluation, the patient was offered foods stained in blue in four consistencies (thin liquid, thickened liquid, semi-solid and solid). Nestlé Resource® food thickener was used to vary the consistency of thin, thickened and semi-solid food, by adding it to water. The patients were offered 5 mL and 10 mL of thin and thickened liquid in a glass, and the semi-solid food was offered in a tablespoon containing 10 mL, whereas the solid food consisted of a 2 × 1 cm wafer biscuit. All exams were recorded on DVD for reanalysis. The swallowing videoendoscopy (SVE) evaluations were followed by two otorhinolaryngologists specialized in laryngeal disorders who followed the examination and consensually defined the score for each item of the examination, without any prior patient information regarding OSA presence, severity and SWAL Qol score. The following parameters were evaluated regarding their presence: 1) Premature oral leakage; 2) Velopharyngeal dysfunction or food nasal escape; 3) laryngeal penetration; 4) tracheal aspiration; 5) presence of residue. Patients with any alteration in the SVE were considered to have dysphagia, with severity being classified as mild when the patient showed premature bolus escape, velopharyngeal dysfunction or presence of food residue, moderate in the occurrence of laryngeal penetration and severe in the case of penetration, as proposed by Macedo Filho et al.^12^

To evaluate the study, the software SPSS, version 21.0, was used for the analyses. Continuous variables are expressed as mean and standard deviation. Categorical variables are expressed as absolute frequencies (n) and percentages (%). The Kolmogorov–Smirnov test was used for data normality analysis. Non-normal data were transformed by Z-score. The General Linear Model test was used for the comparison between groups. Pearson's chi-square test was used to compare the frequency between the groups. The logistic regression model was used to evaluate the predictors for dysphagia, together with the β coefficient and 95% confidence interval (95%CI). The Hosmer–Lemeshow test was used as the test quality criterion. The level of significance was set at α ≤ 0.05.

## Results

All 70 subjects met the study inclusion criteria and spontaneously agreed to participate in the study. The mean age was 48.9 years and 49 were males (70 %). The mean BMI was 31.8 ± 4.6 (SD). The median AHI (Apnea and Hypopnea Index/hour) was 42.4 events / hour (16.3–164.0) and the median RDI (Respiratory Disorder Index) was 45.1 (18.9–117.0). The mean neck circumference was 41.7 ± 4.4 (SD). The means of the anthropometric and polysomnographic parameters are shown in Table 1. The 70 assessed patients were divided into two groups, according to the presence of alterations in the swallowing videoendoscopy (SVE), between the group with dysphagia and the one without dysphagia. Examination abnormalities were observed in 18 patients (27.3 %), who were included in the dysphagia group. Clinical, polysomnographic characteristics and scores in the questionnaires (Swal-QoL, ESS) were compared.

**Table 1:** Anthropometric and polysomnographic parameters of the sample.

	n = 70
Age (years)	48.9 ± 11.2
Gender, n (%)	
Male	49 (70)
Female	21 (30)
Smoking, n (%)	9 (14.1)
Neck circumference (cm)	41.7 ± 4.4
BMI (kg/m^2^)	31.8 ± 4.6
AHI (events/hour)	42.4 (16.3–164.0)
RDI (events/hour)	45.1 (18.9–117.0)
Arousals (arousals/hour)	26.3 (0.0–153.3)
Sleep efficiency (%)	83.0 (49–97.3)
N1 Stage (%)	16.3 (0.9–83.4)
N2 Stage (%)	45.1 (2.3–79.8)
N3 Stage (%)	15.7 (0.0–31.9)
REM stage (%)	15.9 ± 6.5
Minimum SpO_2_ (%)	74.0 (50‒88)
SpO_2_ < 90%	15.5 (0.1–76.1)

Categorical data shown in absolute frequency and percentage; continous data shown as mean and SD.

cm, centimeters; BMI, Body Mass Index; AHI, Apnea-Hypopnea Index; RDI, Respiratory Disorder Index; N1, N2, N3, REM: sleep stages in % of time, SpO_2_min, Minimum O_2_ saturation; SpO_2_ < 90 %, Time in % of O_2_ saturation below 90 %.

Table 2 depicts the descriptive data of the groups with and without dysphagia, which showed a tendency towards statistical significance for the female gender in the presence of dysphagia (*p* = 0.069). There was no statistical difference in the comparison of age, BMI and smoking status in the two groups.

**Table 2:** Comparative analysis of anthropometric characteristics according to the presence of dysphagia.

	With dysphagia (n = 18)	Without dysphagia (n = 48)	*p* value
Age (years)	49.5 ± 11.3	49.3 ± 11.5	0.953
Gender, n (%)			
Male	10 (20.8)	38 (79.2)	0.069
Female	8 (44.4)	10 (55.6)	
Smoking, n (%)	3 (37.5)	5 (62.5)	0.429
Neck circumference (cm)	40.9 ± 4.2	42.1 ± 4.5	0.369
BMI (kg/m^2)^	31.7 ± 4.9	31.9 ± 4.7	0.881

Data shown as mean and SD (±). Data shown in absolute and relative frequency (n, %), *p* < 0.05. cm, centimeters; BMI, Body Mass Index; AHI, Apnea-Hypopnea Index; RDI, Respiratory Disorder Index; N1, N2, N3, REM: sleep stages in % of time, SpO_2_min, Minimum O_2_ saturation; SpO_2_ < 90 %, Time in % of O_2_ saturation below 90 %.

When evaluating the polysomnographic findings, there was no statistical difference related to the severity of obstructive sleep apnea or other parameters evaluated in the polysomnography of patients with and without dysphagia. The comparative analysis between the two groups is shown in Table 3.

**Table 3:** Comparative analysis of polysomnographic characteristics according to the presence of dysphagia.

	Without dysphagia (n = 49)	With dysphagia (n = 18)	*p*
ESS	13.45 ± 5.57	14.76 ± 5.10	0.39
AHI (events/hour)	54.32 ± 31.65	46.48 ± 27.96	0.35
RDI (events/hour)	51.32 ± 26.48	49.72 ± 28.23	0.84
Arousals/hour	35.39 ± 27.72	34.16 ± 26.07	0.87
N1 stage	22.69 ± 19.24	24.09 ± 14.51	0.78
N2 stage	46.82 ± 16.33	43.56 ± 11.85	0.44
N3 stage	14.79 ± 10.16	15.77 ± 9.43	0.72
REM stage	16.28 ± 6.84	15.93 ± 7.19	0.85
O_2_ min	71.40 ± 9.90	74.16 ± 9.59	0.31
% O_2_ < 90%	24.18 ± 23.74	18.78 ± 18.26	0.42

Data shown as mean and SD (±); Univariate GLM Test ≠ Data shown as absolute and relative frequency (n, %); *p* < 0.05, Chi Square Test.

BMI, Body Mass Index; ESS, Epworth Sleepiness Scale; AHI, Apnea and Hypopnea Index; RDI, Respiratory Disorder Index; BAI, Brief Arousal Index; N1, N2, N3, REM, Stages of sleep; SpO_2_min, Minimum O_2_ Saturation; SpO_2_ < 90 %, Time in % O_2_ saturation below 90 %.

**Figure 1:**
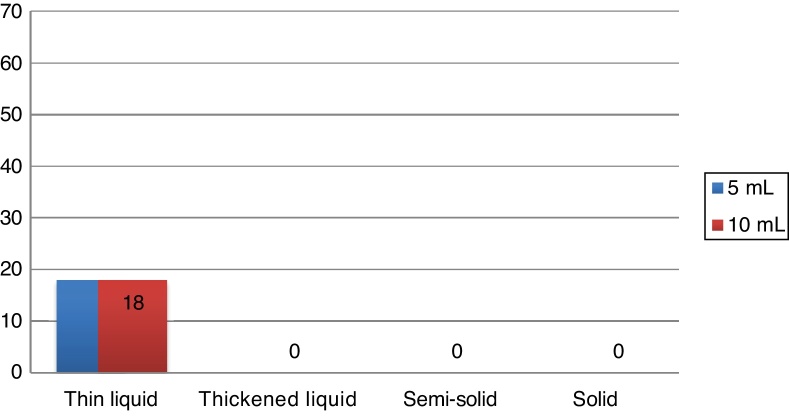
Representation of swallowing alterations at the videoendoscopy in OSA patients according to different tested volumes and textures. Values are shown in absolute frequency.

During the Swallowing Videoendoscopy (SVE), the 18 individuals (27.3 %) who had alterations in the examination showed premature oral leakage with thin liquid, which occurred with the 5 mL and 10 mL samples. The study showed no velopharyngeal dysfunction, laryngeal penetration or tracheal aspiration. In all cases, the presence of food bolus residue in the pharynx was eliminated after three swallows, being considered normal regarding the occurrence of food residues. The alteration found in the SVE of these 18 patients was classified as mild dysphagia, according to previously mentioned criteria. The swallowing videoendoscopy findings are shown in Fig. 1.

As for the dysphagia quality of life questionnaire (Swal-Qol), regarding the total score, there was no difference between the groups without and with dysphagia. However, considering each domain alone, statistical significance was observed in domain two (desire to eat/duration of feeding), with *p* = 0.015; being worse in the group with dysphagia.

## Discussion

Although there are studies in the scientific literature that have shown an increased prevalence of dysphagia in OSA patients, the evaluation of the factors associated with it has been rarely analyzed.

The main objective of the study was to evaluate the clinical and polysomnographic predictors of dysphagia in patients with obstructive sleep apnea; however, it is important to highlight the possibility of other dysphagia-related factors not elucidated in the present study. Regarding the inclusion criteria of the assessed patients, the maximum age limitation of 65 years was intended to minimize the occurrence of presbyphagia, although there is no defined age for the onset of swallowing alterations due to probable physiological adaptations in swallowing throughout life. Regarding the literature, the increased incidence of dysphagia according to age is mainly associated with a higher frequency of diseases potentially related to swallowing disorders in the elderly.

Age, as well as smoking, in the current sample, did not show statistical relevance for the occurrence of dysphagia. Another limiting factor in the current study is that there was no exclusion of patients with pharyngolaryngeal reflux (PLR), a disease that potentially causes chronic upper airway inflammation. The association between obstructive sleep apnea has been discussed in the literature in recent years, but the correlation between them is not a consensus.^14^ This is due to the fact that some studies have shown a positive association between the two diseases and other studies have not found any causality between OSA and PLR. Moreover, some authors suggest that the coexistence of both diseases occurs because they share the same risk factors, such as obesity and gender.^15^

The results shown here confirmed that patients with OSA have subclinical swallowing dysfunction, although this alteration does not result in significant worsening of these patients’ quality of life.

Considering the clinical characteristics of OSA patients who showed alterations in the swallowing videoendoscopy, the presence of dysphagia tended to be statistically significant related to the female gender, but not related to age, BMI and neck circumference. In partial agreement, Valbuza et al. found no association between dysphagia and the individuals’ age or BMI.^7^ Oliveira LAMP et al. did not demonstrate an association between the occurrence of age-related dysphagia, BMI and neck circumference. However, they report that the patients with laryngeal penetration were older.^16^ Also in this study, regarding gender, individuals with dysphagia were predominantly males (70.6 %), although this was not a risk factor for the occurrence of dysphagia. The trend of significance in the association between female gender and dysphagia evidenced in the present study is not yet found in the literature. The increased prevalence of dysphagia in the elderly is well clarified in the literature, related to muscle atrophy and the cognitive decline that occurs with aging.^17^, ^18^ As for gender, studies have shown differences in the swallowing characteristics of healthy individuals of different ages, in which women showed higher pharyngeal muscle reserve and flexibility in the compensatory swallowing mechanism in comparison to men, suggesting that females have a compensatory mechanism for aging, more efficient than that of the male gender.^19^ The female gender, regarding the pathophysiology of obstructive sleep apnea, is less prone to the development of the disease than the male gender, with menopause being a risk factor for the worsening of apnea.^20^ In the current study, a possible explanation for the increased prevalence of females in the dysphagia group could be the association between dysphagia and age and obstructive sleep apnea that occurs at an older age range in women than in men.

Regarding the influence of polysomnographic factors regarding the presence of dysphagia, the present study corroborates literature data, in which OSA severity does not correlate with dysphagia. Schindler et al., who divided their sample into a group with moderate apnea and another with severe apnea, also found no significant difference regarding the occurrence of dysphagia in both groups.^4^ The study by Oliveira et al. also found no association between dysphagia and OSA severity.^16^ In a previous study by Kimoff et al., who evaluated upper airway mucosal sensory dysfunction in OSA patients and in non-apnetic snorers, compared with a control group of non-snorers, in fact did not show any difference between snorers and OSA patients.^21^ These data indicate that trauma caused by tissue vibration, secondary to snoring, causes sensory dysfunction in the pharyngeal mucosa, leading to upper airway neuronal receptor injuries that would contribute to dysphagia. However, the risk of dysphagia apparently does not correlate with OSA severity.^4^, ^16^, ^21^ Considering other aspects of the polysomnographic findings, the existence of dysphagia showed no correlation with the brief arousal index, O_2_ saturation below 90 % during sleep or with sleep architecture. In contrast, a study by Teramoto evaluating 24 patients with OSA *versus* 24 age-matched controls detected changes in the swallowing reflex by administering a distilled water bolus through the nose with a suprapharyngeal pressure transducer catheter and such alterations were related to more frequent oxyhemoglobin desaturation.^5^ In this case, the method for detecting swallowing alterations differs from that in the current study, preventing comparisons on the occurrence of dysphagia between them, but highlighting another possible deleterious mechanism in the swallowing function of OSA patients besides the sensory dysfunction resulting from pharyngeal trauma, as previously elucidated.

Patients with OSA have a subclinical swallowing dysfunction, although this dysfunction is of low severity. In the SVE examination, dysphagia was observed in 27.3 % of cases, with all cases being related to premature oral leakage with thin liquid, both with 5 mL and 10 mL. There were no other SVE alterations in the current study. The absence of a control group without obstructive sleep apnea was a limitation of the current study. In the literature, a study with a control group related to the topic used videofluoroscopy as a diagnostic method of dysphagia, reporting alteration of swallowing with premature leakage of the food bolus into the pharynx and delayed swallowing reflex onset in 54 % of 41 assessed patients, 20 of them with primary snoring and 21 with mild and moderate OSA and only in 7 % of the 15 non-snoring patients in the control group, with statistical difference.^6^ Comparing the findings with those of previous studies that also used SVE, in the current study the prevalence of dysphagia was lower than that found in the literature. Schindler et al. evaluated 72 patients with moderate or severe OSA found 64 % of cases with premature oral leakage with a 20 mL sample.^4^ The difference regarding the literature could be related to SVE protocol variation. In the current study, we chose to maintain the service routine SVE protocol with the 5 mL and 10 mL samples, according to the previous protocol by Santoro et al.,^22^ thus, not overestimating the occurrence of dysphagia in relation to other patients submitted to the swallowing videoendoscopy examination in the service. Comparing the findings of dysphagia with others in the literature, Oliveira LAMP shows that 68.2 % of dysphagia cases are related to premature oral leakage and Valbuza et al. reported 64 % also associated with premature oral leakage. As in the present study, premature oral leakage was considered only when it occurred with liquid or semi-solid consistency, which is a possible reason for the lower prevalence of dysphagia in the study. The option of disregarding premature oral leakage of solids in dysphagia classification was due to the high frequency of premature oral leakage of solid consistency in normal subjects.^23^

In clinical evaluation of patients with OSA, dysphagia-related symptoms are not reported spontaneously, and this is indeed verified in the literature, in which dysphagia is a subclinical disorder in this population. For this reason, the choice of SWAL-QoL questionnaire in the current study was chosen to verify whether the presence of dysphagia in OSA patients, although not clinically evident, could have a negative impact on these patients. However, the SWAL-QOL was not a tool capable of predicting the occurrence of dysphagia in OSA patients, given the absence of statistical difference in the questionnaire scores in the groups with and without dysphagia.

## Conclusion

The occurrence of dysphagia in this group of patients with moderate to severe OSA was 27.3 %, and the most frequent finding was the premature oral leakage. Although the cases show mild dysphagia, the complaint related to any swallowing disorder is not spontaneously reported by patients with obstructive sleep apnea, which supports the need to investigate dysphagic symptoms in these patients.

There was no further worsening of the dysphagia-related quality of life in dysphagic patients when compared to those without dysphagia, suggesting that although present in patients with moderate to severe OSA, the alteration has a slight impact on QoL. This study should be considered as a preliminary assessment of swallowing disorders in sleep apnea patients and further studies are needed to clarify additional factors related to the presence of dysphagia in individuals with apnea, as well as the possibility of swallowing disorders aggravating other comorbidities found in these patients.

## Conflicts of interest

The authors declare no conflicts of interest.
